# Prevalence of postpartum family planning utilization and associated factors among postpartum mothers in Arba Minch town, South Ethiopia

**DOI:** 10.1186/s40834-021-00150-z

**Published:** 2021-03-02

**Authors:** Biresaw Wassihun, Kidist Wosen, Asmare Getie, Kalkidan Belay, Rehal Tesfaye, Tewodros Tadesse, Yosef Alemayehu, Manaye Yihune, Addis Aklilu, Kassahun Gebayehu, Shegaw Zeleke

**Affiliations:** 1 College of Medicine and Health Sciences, Arba Minch University, Arba Minch, Ethiopia; 2 Department of Nursing, College of Medicine and Health Sciences, University of Gondar, Gondar, Ethiopia; 3 Colleges of Medicine and Health Sciences, Debre Tabor University, Debre Tabor, Ethiopia

**Keywords:** Postpartum, Family planning, Utilization, Ethiopia

## Abstract

**Background:**

Contraception allows women to realize their human right to decide if and when to have children and helps people to attain their desired family size. Yet 214 million women of a reproductive age in developing countries who want to avoid pregnancy are not using a modern contraceptive method. Women who have recently given birth are among the group with the highest unmet need for contraception. Therefore, this study was aimed to assess the prevalence of postpartum family planning use and associated factors among postpartum women in Southern Ethiopia.

**Methods:**

Institution based cross-sectional study design was conducted. A structured and pretested interviewer-administered questionnaire was used to collect the data from study participants. Study participants were selected using a systematic random sampling technique by allocating proportionally to each health facility. The data was entered using EPI data version 3.1statistical software and exported to Statistical Package for Social Sciences version 22.0 for further analysis. Both bivariate and multivariate logistic regression analyses were performed to identify associated factors. *P* values < 0.05 with 95% confidence level was used to declare statistica significance.

**Result:**

Overall, 44% of postpartum women utilize postpartum family planning. Having an antenatal care visit [adjusted odds ratio (AOR) =1.89(95%CI, 2.42–7.90), having planned pregnancy [adjusted odds ratio (AOR) = 1.17(95%CI, 1.60–2.28)], being married (adjusted odds ratio (AOR) =2.86(1.94–8.73), and having a college and above level educational status (AOR) =1.66(1.28–3.55) were significantly associated with utilization of postpartum family planning.

**Conclusion:**

This study showed that the prevalence of postpartum family planning was 44%. Marital status, educational status of mothers, the status of pregnancy, and having an antenatal care follow-up during pregnancy were some factors associated with postpartum family planning utilization. Therefore, strengthening family planning counselling during antenatal and postnatal care visits, improving utilization of postnatal care services and improving women’s educational status are crucial steps to enhance contraceptive use among postpartum women.

## Background

Contraception allows women to realize their human rights, and decide if and when to have children, and also helps women to attain their desired family size. Yet 214 million women of the reproductive age group is in developing countries who want to avoid pregnancy are not using a contraceptive method. Women who have recently given birth are amongst those with the highest unmet need for contraception [1, 2].

Studies show that roughly 95% of women who are 0 to 12 months postpartum want to avoid pregnancy in the next 24 months, but 70% of them are not using contraception. Pregnancies in the postpartum period pose the greatest risk for women, and their infants, and have increased risks of adverse health outcomes [1, 2]. To reduce the risk of adverse maternal, perinatal, and infant outcomes, the World Health Organization (WHO) recommended that the interval between live birth, and an attempt to the next pregnancy should be a minimum of two years [2]. By providing postpartum family planning it is possible to reduce maternal and child mortality [3]. Women need to avoid pregnancy so soon after delivery but due to different reasons, they could not be able to get contraceptives [4]. Postpartum family planning has a dual advantage to reduce maternal mortality and to reduce population growth in low resource countries [5]. The integration of family planning as the part of a comprehensive public health approach is important to achieve sustainable development goals [6]. According to Ethiopian demographic health surveillance (EDHS), 4 and 9% of pregnancies occur within less than six months and less than twelve months respectively after prior delivery [7]. This closely spaced birth interval has a potential effect on the increased risk of maternal death [8]. An increase in contraceptive use during the postpartum period significantly reduces the rates of maternal and infant mortality by averting unplanned and unwanted pregnancies and spacing new pregnancies to at least two years after the previous birth. Moreover, the largest proportion of women with an unmet need for contraception is found amongst those in their first year after childbirth [9, 10]. The finding of the previous study showed that the majority of women would prefer to avoid becoming pregnant within two years after delivery, yet only 40% are using a family planning method worldwide [4]. The majority of maternal and child death in Ethiopia was due to pregnancy and its related complications [11]. One of the goals of the ministry of health of Ethiopia, concerning improving maternal and child health, is to raise the contraceptive prevalence rate to 66% by 2015. Currently, overall family planning coverage and modern contraceptive uses that are the key indicator of maternal and child health remain low in the country 29 and 27%, respectively [12]. Despite the accepted demand for postpartum contraception, many postpartum women do not have access to family planning information or service that they need to delay or prevent consequent pregnancy [13]. A previous study, which was conducted in Ethiopia, showed that the prevalence of postpartum family planning was low as compared to other countries. There is no study conducted in our study area on the utilization of postpartum family planning and associated factors so this study fills this gap.

## Methods

### Study area and period

This study was conducted in public health facilities in Arba Minch town, which is the administrative town of the Gamo zone in South Ethiopia. Gamo zone is among the 15 zones found in the Southern Nations, Nationalities, and Peoples Region. Arba Minch town is located at a distance of 495 km from Addis Ababa and 275 km from Hawassa, the capital of Southern Nations, Nationalities, and Peoples Region in the South direction. There is one Governmental hospital (Arba Minch general hospital) and two public health centers. The study was conducted in all health facilities between October 2018 and January 2019.

### Study design

Health facility-based cross-sectional study was conducted.

### Study population

The study participants were postpartum women who came for postnatal care (after six weeks of.

Postpartum).

### Sampling criteria

#### Inclusion criteria

Those postpartum women who delivered at least before six weeks were included in the study.

#### Exclusion criteria

Mothers who were critically ill and unable to communicate during the data collection period.

### Sample size determination

The sample size required for this study was calculated based on a single population proportions formula with the following assumptions: n is a sample size, Z is standard normal distribution corresponding to significance level at α = 0.05, d is a margin of error assumed to be 5%, P is anticipated proportion of women who had used post-partum family planning 41% taken from the previous study. With the above inputs, the minimum sample size required for the study is 371 taking a 10% non-response rate the final sample size was 408.

### Sampling procedure

**Fig. 1:**
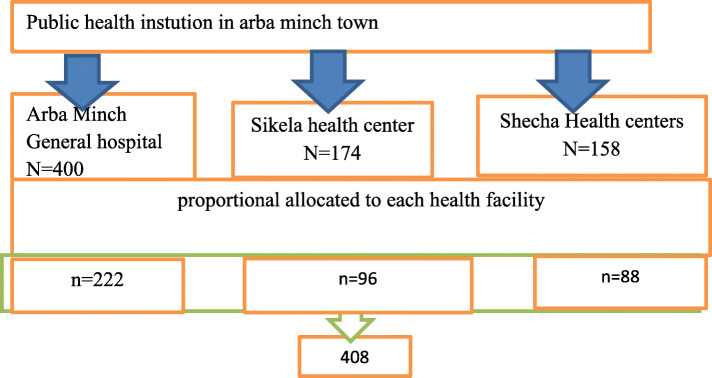
Flow chart indicating the sampling procedure of postpartum family planning utilization in Arba Minch Tow

There is one public hospital (Arba Minch General Hospital) and two public health centers (Sikela and Shecha Health centers) in Arbaminch town and all the health institutions were included in the study. The average number of children who received Vaccination in Arbaminch hospital, Sikela health center, and Shecha health center is 400, 174, and 158 respectively. To include 408 in the study, the proportional allocation method was done which means 222 women from the Arba Minch hospital, 96 women from the Sikela health center, and 88 women from Shecha health center. Data collector use systematic sampling technique to interview the respondents by considering the assumption of **N** (the average number of children who received Vaccination three months period in the health institutions which is 732, and **n** (required minimum sample size = 408 which gives a k of 2): K = N/n = > 732/408 = 1.8 ≈ 2 (Fig. 1).

### Data collection tool

Data were collected by face-to-face interviews using a structured questionnaire adapted from the different literature. The questionnaire was used to assess the post-partum family planning utilization and associated factors. The entire questioner was checked for completeness and accuracy before and after the period of data collection. Four BSc Midwifery students who were fluent in speaking the local language were involved in the data collection. The principal investigator supervised the data collection process.

### Data quality control

A structured interviewer-administered questionnaire was first prepared in English and then translated into local languages, then back to English by independent language experts to ensure its consistency. The explanation was given to data collectors about techniques of data collection and briefed on each question included in the data collection tool by principal investigators. After that, a pre-test was conducted on 5% of the sample size outside the study area before 15 days of actual data collection to ensure the validity of the tool, then correction was made accordingly.

### Data processing and analysis

The collected data were checked visually by the investigators, then data were coded, entered, and cleaned using Epi-Data version 3.1 software and finally exported into SPSS version 22 for analysis. Descriptive statistical analysis such as simple frequencies, measures of central tendency, and measures of variability was used to describe the characteristics of participants. Then the information was presented using frequencies, summary measures, tables, and figures (charts). Bivariate analysis, COR with 95% CI, was used to see the association between each independent variable and the outcome variable by using binary logistic regression. Independent variables with a *p*-value of ≤0.25, significant in previous st.

udies, and based on the context were included in the multivariable analysis. Multi-collinearity was checked to see the linear correlation among the independent variables by using the variance inflation factor and standard error. Variables with variance inflation factor > 10 and standard error of > 2 were dropped from the multivariable analysis. AOR with 95% CI was estimated to identify the factors associated with post-partum family planning using multivariable logistic regression analysis. The level of statistical significance was declared at p-value ≤0.05.

## Result

### **Socio-demographic characteristics of mother**s

From a total of 408 respondents who were invited for an interview, all consented to participate in the study giving a response rate of (100%). The mean age of the respondents was 26.8 (SD ± 5.5) years with a minimum and maximum age of 16 and 42 respectively. Almost half of the study participants 201 (49.4%) were from the Gamo ethnic group and 206 (50.5%) were Orthodox Christianity religious followers. Regarding their marital status, 345 (84.6%) of them were married. Out of the total respondents, 244 (59.8%) of them had a monthly family income less than or equal to 107 USD. And the majority of mothers, 148(36.3%) had secondary education followed by 117(28.7%) college and above (Table 1).

**Table 1:** Socio-demographic characteristics of respondents in Arba Minch town public health facility, South, Ethiopia, 2019 (*n* = 408)

Variables	Frequencies	Percent
**Age**		
15–20	55	13.5
21–25	260	63.7
26–30	59	14.5
31 and above	34	8.3
**Marital status**		
Married	345	84.6
Divorced	15	3.7
Widowed	19	4.7
Single	29	7.1
**Ethnicity**		
Gamo	201	49.5
Gofa	73	17.9
Amhara	61	15.0
Waleyta	56	13.7
Others	17	4.2
**Educational status of mothers**		
No formal education	39	9.6
Primary	104	25.5
Secondary	148	36.3
College and above	117	28.7
**Respondents religion**		
Orthodox	206	50.5
Muslim	58	14.2
Catholic	2	0.5
Protestant	142	34.8
**Mothers residency**		
Urban	348	85.3
Rural	60	14.7
**Mothers occupation**		
Housewife	177	43.4
Private employee	46	11.3
Government employee	93	22.8
Merchants	78	19.1
Daily workers	14	3.4
**Family monthly income**		
≤ 107USD	244	59.8
> 107USD	164	40.2

### Obstetric history of respondents

Out of 408 respondents, 365 (89.5%) had a history of antenatal care follow up. The majority of 257 (72%) of the respondents were multigravida and 236 (57.9%) were multipara. From the total of respondents, 349 (85.5%) of the pregnancy was planned and supported and almost all of the respondents 334 (81.8%) of the respondents had no previous history of abortion and also 349 (85.5%) of the respondents had no history of stillbirth **(**Table 2).

**Table 2:** Obstetric characteristics of respondents in Arba Minch town public health facility, South, Ethiopia, 2019 (*n* = 408)

Variables	Frequencies	Percent
**ANC**		
Yes	365	89.5
No	43	10.5
**Institutional delivery history**		
Yes	356	87.3
No	52	12.7
**Parity**		
One	172	42.2
Two	139	34.1
Three and above	97	23.8
**Status of pregnancy**		
planned	349	85.5
Un planned	59	14.5
**History of abortion**		
Yes	74	18.1
No	334	81.8
History of stillbirth		
Yes	58	14.3
No	349	85.5

### Utilization of family planning

**Fig. 2:**
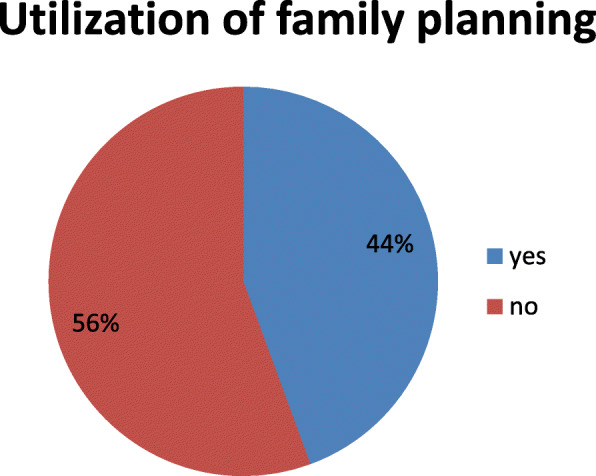
Overall current utilization of postpartum family planning in Arba Minch town Southern Ethiopia, 2019 (*n* = 408)

From the total of 408 respondents, 181(44.4%) use post-partum family planning whereas 227(55.6%) didn’t use postpartum family planning, majority of 70(38%) use implant as post-partum family planning followed by oral pill 47(25%) and 24(13.2%) of the respondents use injection(depo) (Fig. 2 and Table 3).

**Table 3:** Utilization of family planning methods in Arba Minch town Southern Ethiopia, 2019(*n* = 408)

Variable	Frequencies	Percent
Currently, the use of family planning	181	44.4
**Family planning method used**		
Oral pill	47	25.9
Emergency pill	11	6.1
Condom	8	4.4
IUCD	21	11.6
Implant	70	38.7
Injection	24	13.3

### Source of information for postpartum family planning

Out of 408 respondents, 394(96.5%) heard information about post-partum family planning. Of those who heard family planning information majority of 327 were heard from health institution, 11 from family, 45 from friend and relatives, and 11 from mass media of those who heard family planning majority of them heard oral contraceptives as a family planning 242(59.7%), IUCD 19(4.8%), injections 43(10.9%), **(**Table 4).

**Table 4:** Source of information towards Postpartum contraceptive in Arbaminch town, Southern Ethiopia 2019(*n* = 408)

Variables	Frequencies	Percent
Heard of contraceptive	394	96.5
**Source of information**		
From health institution	327	82.9
From family	11	2.79
Friend	45	11.4
Mass media	11	2.79
**Which method do you know**		
Oral pill	242	59
Emergence pill	21	7.8
Condom	69	17.5
IUCD	19	4.8
Injection	43	10.9

### Factors associated with family planning utilization

The result of multiple logistic regression analysis showed that respondent educational status, marital status. Having antenatal care follow up and status of pregnancy were some of the factors associated with post-partum family planning utilization at *P*-value < 0.05. Respondents who have had the educational status of college and above were 1.66 times more likely to utilize family planning than respondents who had no formal education [adjusted odds ratio (AOR) = 1.66(95%CI; 1.28,3.55)]. Similarly, respondents having the marital status of married were 2.86 times more likely to utilize postpartum family planning than respondents who had single [adjusted odds ratio (AOR) = 2.86(95%CI; 1.94, 8.73)]. Respondents who had planned pregnancy were 1.17 times more likely to utilize post-partum family planning than respondents who had unplanned pregnancy [adjusted odds ratio (AOR) = 1.17(95%CI; 1.60,2.28)] (Table 5).

**Table 5:** Bivariate and multivariate logistic regression analysis with its predictor variables in Arbaminch town, Southern Ethiopia, 2019(*n* = 408)

Variable	Family planning utilization		Crud odds ratio COR (95%CI)	Adjusted odds ratio AOR(95%CI)
	Yes	No		
**Residence**				
Urban	159	189	1.	1
Rural	22	38	1.45 (0.82–2.55)	0.84 (0.45–1.58)
**Marital status**				
Marred	161	184	3.35 (1.33–8.44)*	2.86 (1.94–8.73)*
Divorced	6	9	1.31 (0.45–3.76)	1.23 (0.41–3.69)
Widowed	8	11	1.20 (0.47–3.06)	1.15 (0.42–3.12)
Single	6	23	1	1
**Educational status of mothers**				
No formal education	14	25	1	1
Primary	39	65	0.93 (0.43–2.55)	0.98 (0.43–2.22)
Secondary	71	77	0.58 (0.29–1.26)	0.67 (0.30–1.53)
College and above	57	60	1.58 (1.27–3.24)*	1.66 (1.28–3.55)*
**Educational status of Husband**				
No formal education	9	21	2.22 (1.97–5.04)*	1.58 (0.60–4.11)
Primary	26	32	1.17 (0.65–2.08)	0.87 (0.44–1.73)
Secondary	29	47	1.54 (0.90–2.61)	1.41 (0.80–2.49)
College and above	116	187	1	1
**Type of pregnancy**				
Planned	162	187	1.82 (1.01–3.27)*	1.17 (1.60–2.28)*
Un planned	19	40	1	1
**History of ANC**				
Yes	166	199	1.55 (1.80–3.01)*	1.89 (2.42–7.90)**
No	15	28	1	1
**Income**				
< 107USD			1.05 (0.70–1.56)	0.84 (0.53–1.32)
≥ 107USD			1	`1

* indicates *p* <0.05

** indicates *P* ≤0.01

## Discussion

The finding of this study revealed that the prevalence of post-partum family planning utilization was 44%. This finding is lower than a study conducted in Burkina Faso (56%), Malawi (75.5%), and Kenya (52.1%) [3, 14, 15]. The discrepancy might be due to socio-economic and cultural differences. On the other hand, the finding was higher than studies that were done in Burundi (20%), Uganda (28%), Kebribeyah Town Ethiopia (12.3%), and Butajira District South Central Ethiopia, 25% [16–19]. The discrepancy might be due to differences in sample size because the previous study used a large sample size. Besides, it might be due to the differences in socio-demographic characteristics and types of data used in which the former studies used secondary data but the data for our study was primary. The finding of this study was consistent with a study conducted at Gondar town, Ethiopia (48.4%) [20].

Respondent educational status, marital status. Having antenatal care follow up and status of pregnancy were some of the factors associated with post-partum family planning utilization at *P*-value < 0.05.

In this study, married mothers were 2.8 times more likely to use postpartum modern contraceptives. This is in line with the study done at Addis Ababa [11]. This might be due to since married women living with their husbands, might start regular sexual intercourse earlier than the non-married couples that may necessitate the utilization of PPFP to program the birth of the next child.

ANC utilization was the other important variable affecting contraceptive use. Women who had antenatal care follow up during pregnancy were 1.89 times more likely to use postpartum family planning than others. The possible explanation is women who attend antenatal care are more likely to get information about contraceptive use. This is consistent with a prospective study done in Kenya and Zambia [20, 21].

In this study, respondents who have had the educational status of college and above were 1.66 times as likely to utilize FP methods as illiterate participants were. This finding implies a significant role in the education status of participants in utilizing available health care services. Education status was reported as a predictor of the use of family planning methods by a few other studies as well [22, 23]. Similarly higher education level attainment invariably gives postpartum women a better understanding of the available modern contraceptive methods and the benefits of fertility regulation and hence the need for contraception during the postpartum period. Besides, higher education increases awareness of the side effects of contraceptive methods and preference for the most convenient ones [16]. The finding of this study showed that mothers who had planned pregnancies were 1.17 times more likely to use postpartum family planning than others. This finding was similar to other study done in Ethiopia [16, 19].

## Conclusions

The finding of this study showed that the prevalence of post-partum family planning utilization was low as compared to others’ study and some of the identified factors associated with post-partum family planning utilization were being married, higher education level, having antenatal car follow up, and having planned pregnancy. Providing health education is an important step to improve post-partum family planning utilization in collaboration with various family planning stakeholders. Mothers should be addressed not only during antenatal care checkup days but also in immunization clinics, mother group meetings, and during home visits by health extension workers. Incorporation of family planning services with maternal and child health (MCH) should continue highly strengthened is recommended to increase contraceptive use in the post-partum period in Ethiopia. Mobilization of the community is also important because the responsibility to reduce maternal & infant mortality through the promotion of family planning lies not only in the medical profession alone but also on the social and political leaders.

### Limitation of the study

- The limitation of this study includes; the present study focused on mothers only but
  better to include institutions delivering service, health care providers, and male partners to identify factors influencing utilization of postpartum contraception
- lack of qualitative study to dig out psychosocial factors that hindering the utilization of family planning.
- This study was conducted in selected government hospitals; hence the findings might not adequately reflect the entire population.

## Data Availability

The datasets used and/or analyzed during the current study available from the corresponding author on reasonable request.

## Abbreviations

ANC: Antenatal care
AOR: Adjusted odds ratio
CI: Confidence interval
PPFP: Postpartum family planning
